# Cap-Specific m^6^Am Methyltransferase PCIF1/CAPAM Regulates mRNA Stability of *RAB23* and *CNOT6* through the m^6^A Methyltransferase Activity

**DOI:** 10.3390/cells13201689

**Published:** 2024-10-12

**Authors:** Ai Sugita, Ryoya Kano, Hiroyasu Ishiguro, Natsuki Yanagisawa, Soichiro Kuruma, Shotaro Wani, Aki Tanaka, Yoshiaki Tabuchi, Yoshiaki Ohkuma, Yutaka Hirose

**Affiliations:** 1 Department of Gene Regulation, Faculty of Pharmaceutical Sciences, University of Toyama, 2630 Sugitani, Toyama 930-0194, Japan; ai_sugita@kirin.co.jp (A.S.); d2362303@ems.u-toyama.ac.jp (R.K.); hr.ishiguro@gmail.com (H.I.); umviera.20love@gmail.com (N.Y.); kurusou.giga@icloud.com (S.K.); swrpap2@gmail.com (S.W.); atanaka@pha.u-toyama.ac.jp (A.T.); ohkumay@mocha.ocn.ne.jp (Y.O.); 2 Division of Molecular Genetics Research, Life Science Research Center, University of Toyama, 2630 Sugitani, Toyama 930-0194, Japan; ytabu@cts.u-toyama.ac.jp; 3 Department of Biochemistry, Graduate School of Biomedical Sciences, Nagasaki University, 1-12-4 Sakamoto, Nagasaki 852-8523, Japan

**Keywords:** gene expression, mRNA modifications, mRNA stability, transcription, mRNA cap, m^6^A, m^6^Am, PCIF1

## Abstract

Chemical modifications of cellular RNAs play key roles in gene expression and host defense. The cap-adjacent *N*^6^,2′-*O*-dimethyladenosine (m^6^Am) is a prevalent modification of vertebrate and viral mRNAs and is catalyzed by the newly discovered *N*^6^ methyltransferase PCIF1. However, its role in gene expression remains unclear due to conflicting reports on its effects on mRNA stability and translation. In this study, we investigated the impact of siRNA-mediated transient suppression of PCIF1 on global mRNA expression in HeLa cells. We identified a subset of differentially expressed genes (DEGs) that exhibited minimal overlap with previously reported DEGs. Subsequent validation revealed that PCIF1 positively and negatively regulates RAB23 and CNOT6 expression, respectively, at both the mRNA and protein levels. Mechanistic analyses demonstrated that PCIF1 regulates the stability of these target mRNAs rather than their transcription, and rescue experiments confirmed the requirement of PCIF1’s methyltransferase activity for these regulations. Furthermore, MeRIP-qPCR analysis showed that PCIF1 suppression significantly reduced the m^6^A levels of *RAB23* and *CNOT6* mRNAs. These findings suggest that PCIF1 regulates the stability of specific mRNAs in opposite ways through m^6^A modification, providing new insights into the role of m^6^Am in the regulation of gene expression.

## 1. Introduction

Over 160 diverse chemical modifications have been identified in cellular RNAs [1]. Some of these modifications were reported to have significant roles in the regulation of the function and metabolism of modified RNAs and in the discrimination between self and non-self RNAs [2,3,4]. *N*^6^-methyladenosine (m^6^A) is the most abundant internal modification in eukaryotic mRNA, and its dynamic deposition on mRNA plays critical roles in most steps of mRNA metabolism, including transcription, processing, degradation, and translation [5,6,7]. Through these regulatory functions, m^6^A is involved in various biological processes such as development, stress responses, immunity, and disease [3,8,9,10,11].

In addition to the internal m^6^A modification, adenosine at the transcription start site following the m^7^G cap structure is also frequently methylated at the *N*^6^ position in vertebrate mRNAs, forming *N*^6^,2′-*O*-dimethyladenosine (m^6^Am) [12,13]. This cap-specific m^6^Am is found in 10–50% of mammalian mRNAs [13,14] and is formed by the sequential action of two distinct RNA methyltransferases, CMTR1 (cap methyltransferase 1) and PCIF (phosphorylated CTD interacting factor 1). CMTR1 methylates ribose 2′-*O* in the first transcribed nucleotide, and when it is adenosine, its *N*^6^ position is further methylated by PCIF1, also known as cap-specific adenosine methyltransferase (CAPAM) [15,16,17,18,19]. The *N*^6^ methyl group of m^6^Am can be removed by FTO (fat mass and obesity-associated protein) demethylase [20,21,22]. Due to its abundance and dynamic nature, m^6^Am is expected to play an important role in regulating gene expression [23].

As PCIF1 depletion completely abolishes cap-specific m^6^Am modifications, PCIF1 is the sole catalytic enzyme responsible for m^6^Am formation [16,18,19,24]. Therefore, multiple studies have been conducted to elucidate the function of m^6^Am in gene expression using PCIF1 knockout cells. However, conflicting results have been reported regarding the role of m^6^Am in mRNA stability and translation. Although Akichika et al. reported that m^6^Am promotes the translation of a subset of mRNAs, Sendinc et al. reported that m^6^Am represses translation [16,19]. Although these groups showed that m^6^Am did not affect mRNA stability, Boulias et al. and Pandey et al. reported that m^6^Am contributes to the stabilization of mRNAs without affecting mRNA translation [18,24]. Therefore, the precise role of m^6^Am in gene regulation remains debatable. These controversial results may be due to differences in the global mapping method for m^6^Am sites and the cell types used. Moreover, the conflicting results may also be attributed to the indirect effects of PCIF1 depletion or unidentified epigenetic changes that may have occurred during the establishment of PCIF1 knockout cell lines. Therefore, to explore the precise functions of m^6^Am in the regulation of gene expression, it is important to examine the effects on gene expression within a shorter time frame after PCIF1 loss.

In this study, we investigated the effect of transient PCIF1 suppression on global mRNA expression in HeLa cells using small interfering RNA (siRNA)-mediated knockdown. Our findings revealed a unique set of differentially expressed genes (DEGs) that exhibited minimal overlap with previously reported DEGs under their PCIF1 knockout conditions. Our subsequent analysis to eliminate the potential off-target effects of siRNAs identified *RAB23* and *CNOT6* as plausible targets of PCIF1. We found that the mRNA expression of *RAB23* increased, whereas that of *CNOT6* decreased upon PCIF1 suppression in four distinct human cell lines. Mechanistic analyses demonstrated that PCIF1 regulated the expression of both targets at the mRNA stability level rather than at the transcriptional level. Rescue experiments have shown that methyltransferase activity of PCIF1 is necessary for this regulation. Finally, MeRIP-qPCR analysis revealed that PCIF1 suppression resulted in a significant reduction in m^6^A levels of these target mRNAs. Taken together, we conclude that PCIF1 regulates the stability of *RAB23* and *CNOT6* mRNAs in opposite ways through m^6^A modification.

## 2. Materials and Methods

### 2.1. Cell Culture

Cells were maintained at 37 °C in a humidified atmosphere containing 5% CO_2_. HeLa S3 cells were cultured in Dulbecco’s Modified Eagle Medium (Shimadzu Diagnostics Corporation, Tokyo, Japan) supplemented with 5% calf serum (Cytiva, Tokyo, Japan), penicillin, streptomycin, and 2 mM L-glutamine. HEK293T, MCF7, and Huh7 cells were cultured in Dulbecco’s Modified Eagle Medium (Shimadzu Diagnostics Corporation) supplemented with 10% fetal bovine serum (Nichirei Biosciences Inc., Tokyo, Japan), penicillin, streptomycin, and 2 mM L-glutamine.

### 2.2. siRNA-Mediated PCIF1 Knockdown

siRNA-mediated PCIF1 knockdown was performed as previously described [25]. The two double-stranded RNA oligonucleotides used for hPCIF1 knockdown (siPCIF1#1 and siPCIF1#3) and a non-targeting negative control RNA were purchased from Thermo Fisher Scientific (Waltham, MA, USA). Another double-stranded RNA oligonucleotide, siPCIF1#2, was designed by Sigma. Transfection was performed using ViaFect™ Transfection Reagent (Promega Corporation, Madison, WI, USA) or Lipofectamine™ RNAiMAX Transfection Reagent (Thermo Fisher Scientific) for DNA and siRNA, respectively.

The sequences of each strand of the siPCIF1#1, siPCIF1#2, and control siRNA (siNC) were previously described [25]. The sequences of each strand of the siPCIF1#3 were as follows:

siPCIF1#3: sense strand: 5′-AAACAUGGAAGGUGACACGACUGGU-3′

antisense strand: 5′-ACCAGUCGUGUCACCUUCCAUGUUU-3′

### 2.3. DNA Microarray Analyses

DNA microarray analyses were performed as previously described [26]. RNA was prepared using a NucleoSpin column kit (Takara Bio Inc., Shiga, Japan). RNA (500 ng) was treated according to the manufacturer’s instructions and subjected to gene expression analysis using a Human Genome U133 Plus 2.0 Array (Thermo Fisher Scientific), followed by characterization using GENESPRING software version 11.0 (Agilent, Santa Clara, CA, USA).

### 2.4. Immunoblotting

Immunoblotting was performed as previously described. The antibodies used were as follows: anti-b-actin (Sigma-Aldrich, Inc., St. Louis, MO, USA, A5441), anti-RAB23 (Proteintech Group, Inc., Rosemont, IL, USA, 11101-1-AP), and anti-CNOT6 (Cell Signaling Technology, Inc., Danvers, MA, USA, #13415). An anti-PCIF1 antibody has been developed in our laboratory [27].

### 2.5. ChIP Assay

The ChIP assay was performed as previously described [25]. The antibodies used were as follows: anti-PCIF1 antibody (developed in our laboratory [27]), anti-Pol II antibody (Santa Cruz Biotechnology, Inc., Dallas, TX, USA, sc-899), and normal rabbit IgG (Medical & Biological Laboratories Co., Tokyo, Japan, PM035).

### 2.6. RT-qPCR

Total RNA was isolated from cells using a NucleoSpin RNA II Kit (Takara Bio Inc). First-strand cDNA was synthesized from total RNA using a PrimeScript™ II 1st strand cDNA Synthesis Kit (Takara Bio Inc.) with random hexamer primers. cDNA was quantified using SYBR^®^ Premix Ex Taq™ II (Takara Bio Inc.) and a Mx3000P Real-Time PCR System (Agilent). Relative RNA expression levels were calculated using the 2^−ΔΔCt^ method and normalized to 18S rRNA.

### 2.7. mRNA Stability Assay

HeLa cells were treated with a negative control siRNA (siNC) and two types of PCIF1-specific siRNAs for 72 h, and 5 µg/mL actinomycin D (Sigma-Aldrich, Inc.) was added to inhibit transcription. Cells were harvested at 0, 2, 4, 8, and 12 h after treatment, and total RNA was isolated. The amount of residual target gene mRNA was measured by RT-qPCR. The relative value was calculated using the expression level of β-actin mRNA (ACTB) as a normalizer.

### 2.8. MeRIP-qPCR

HeLa cells were transfected with siRNA for three days. Total RNA was isolated from the cells using Sepasol™-RNA I Super G (Nacalai Tesque, Inc., Kyoto, Japan) according to the manufacturer’s instructions. An amount of 1.5 μg of anti-m^6^A antibody (Abcam Limited, Cambridge, UK, ab151230) or normal rabbit IgG (Medical & Biological Laboratories Co.) was incubated with 30 μL of Dynabeads Protein G (Thermo Fisher Scientific) at room temperature for 1 h. Total RNA (50 μg) was added to the antibody-Dynabead complexes and incubated at 4 °C for 4 h. After washing three times, the bound RNA was purified from the beads using Sepasol™-RNA I Super G. The first strand of cDNA was synthesized from m^6^A-RNA using ReverTra Ace™ qPCR RT Master Mix with gDNA Remover (Toyobo Co., Osaka, Japan) with random hexamer primers according to the manufacturer’s instructions. cDNA was quantified using GeneAce SYBR™ qPCR Mix II (Nippon Gene Co., Tokyo, Japan) and a Mx3000P real-time PCR system (Agilent).

### 2.9. Primers

The sequences of primers used in this study are shown in the Supplemental Materials S1.

### 2.10. Statistical Analysis

Data are expressed as mean ± standard deviation of at least three independent experiments. Statistical differences between groups were evaluated using Student’s *t*-test. Statistical significance was set at *p* < 0.05.

## 3. Results

### 3.1. Exploration of PCIF1 Target Genes by Genome-Wide Gene Expression Analysis

**Figure 1:**
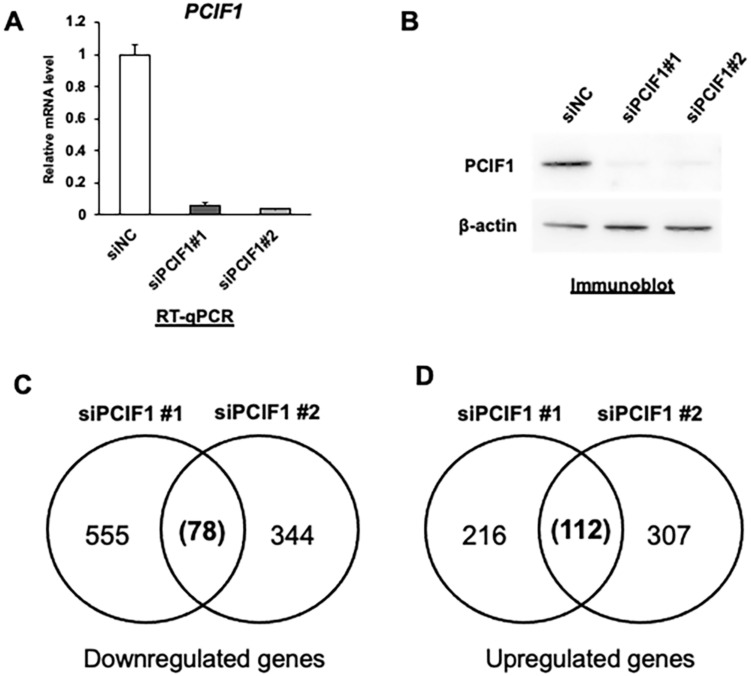
Exploration of PCIF1 target genes by genome-wide gene expression analysis. (**A**) RT-qPCR analysis of total RNAs isolated from HeLa cells treated with control siRNA (siNC) or two distinct PCIF1-targeted siRNAs (siPCIF1 #1 and #2). (**B**) Immunoblotting analysis of total protein extracts from HeLa cells treated with control siRNA (siNC) and two distinct PCIF1-targeted siRNAs (siPCIF1 #1 and #2) with anti-PCIF1 and anti-b-actin antibodies. (**C**,**D**) Venn diagrams showing the overlap between the two indicated siRNA-mediated downregulated (**C**) and upregulated (**D**) genes identified by the gene expression profile analyzed by DNA microarray using a Human Genome U133 Plus 2.0 Array (Affymetrix). The condition for selecting differentially expressed genes are as follows: (1) cut-off condition: expression > 100, (2) fold change: >2, (3) *p*-value < 0.05.

To identify genes whose expression was affected by transient suppression of PCIF1, we performed genome-wide gene expression analyses in HeLa cells transfected with either control or PCIF1-targeting siRNA. Transfection of two different siRNAs targeting PCIF1 (siPCIF1#1 and siPCIF1#2) into HeLa cells significantly reduced PCIF1 expression at both the RNA and protein levels compared to transfection with the negative control siRNA (siNC) (Figure 1A,B). By comparing the mRNA expression profiles determined by DNA microarray analyses between PCIF1 knockdown and control cells, we identified a subset of differentially expressed genes (DEGs) that showed a two-fold or greater change in expression upon PCIF1 knockdown. Among the affected genes, 78 downregulated and 112 upregulated genes were common to each siRNA-treated HeLa cell line (Figure 1C,D).

To explore the cellular pathways regulated by PCIF1, we analyzed DEGs identified by DNA microarray analysis using the Ingenuity Pathway Analysis (IPA) tool. Based on the *p*-values, the top two molecular and cellular function categories for downregulated genes were classified into “Cellular Movement” and “Lipid Metabolism” (Table 1, top), and the upregulated genes were classified into “Molecular Transport” and “Nucleic Acid Metabolism” (Table 2, top). In the physiological system development and function category, the downregulated genes were classified into “Tissue Morphology” and “Hematological System Development and Function” (Table 1, bottom), and the upregulated genes were classified into “Hematological System Development and Function” and “Immune Cell Trafficking” (Table 2, bottom). These results imply that PCIF1 may regulate the expression of a subset of genes associated with metabolic and hematological pathways.

### 3.2. RAB23 and CNOT6 Are the Target Genes of PCIF1

Subsequently, to validate the DNA microarray results, we selected twenty-two genes from the DEGs (thirteen upregulated and nine downregulated genes) and compared their expression between PCIF1 knockdown and control cells using RT-qPCR (Supplementary Figures S1 and S2). Based on the results of the expression changes induced by PCIF1 knockdown using two siRNAs, we further selected eight genes whose expression changes were well correlated with the efficiency of PCIF1 suppression. These genes included upregulated *EGR1*, *CA12*, *RAB23*, *PTGES*, and *HMOX1* and downregulated *PRKA*, *CNOT6*, and *SLC2A3*. To validate these genes as PCIF1 targets, we used a third siRNA targeting *PCIF1* (siPCIF1#3) to eliminate the potential off-target effects of siRNAs and assessed whether it caused similar changes in expression levels. Among the upregulated genes, the expression level of *RAB23* was inversely correlated with the PCIF1 suppression efficiency (Supplementary Figure S3). Among the downregulated genes, the expression levels of *CNOT6* were most significantly correlated with the efficiency of PCIF1 suppression (Supplementary Figure S4). Therefore, we analyzed the regulation of these genes by PCIF1 in detail. In the subsequent analysis, we used two different siRNAs targeting PCIF1, siPCIF1#1 and siPCIF1#3, to suppress the PCIF1 expression because the other, siPCIF1#2, sometimes caused an extreme effect on the expression of several genes (Supplementary Figure S3), and we suspect an unknown off-target effect.

**Figure 2:**
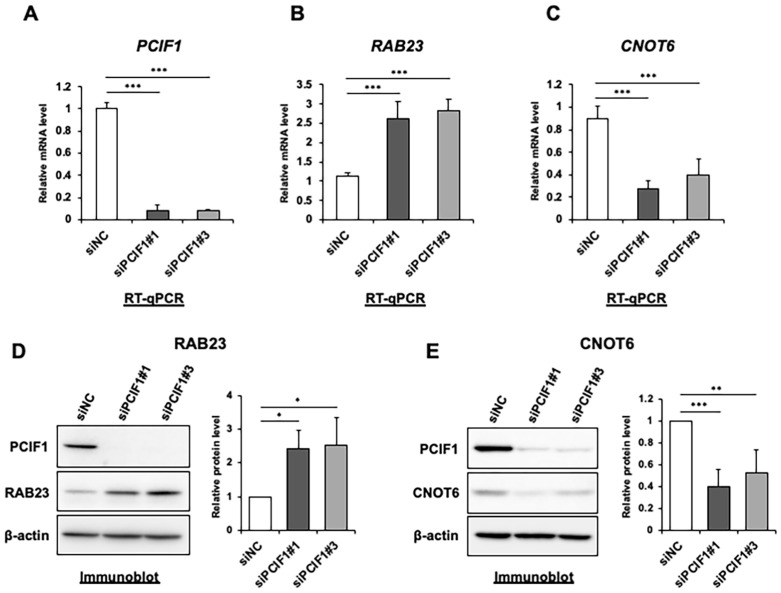
Expression of *RAB23* and *CNOT6* is regulated by PCIF1 at both the mRNA and protein levels. (**A**–**C**) RT-qPCR analysis of total RNAs isolated from HeLa cells treated with control siRNA (siNC) and two distinct PCIF1-targeted siRNAs (siPCIF1 #1 and #3), using the specific primer set detecting PCIF1 (**A**), *RAB23* (**B**), and *CNOT6* (**C**) expression. (**D**,**E**) Immunoblotting analysis of total protein extracts from HeLa cells treated with control siRNA (siNC) and two distinct PCIF1-targeted siRNAs (siPCIF1 #1 and #3) with the indicated antibodies. Signal intensities obtained from immunoblotting were quantified using ImageJ software version 1.52. The y-axis represents the fold change relative to the levels in HeLa cells treated with control siRNA. Data are expressed as the mean ± standard deviation of three independent experiments. Asterisks represent statistically significant differences between the control siRNA treatment and the indicated PCIF1-targeted siRNA (Student’s *t*-test, * *p* < 0.05, ** *p* < 0.01, *** *p* < 0.001).

We first examined whether the protein expression levels of RAB23 and CNOT6 are also altered by PCIF1 suppression. PCIF1 knockdown in HeLa cells using two different siRNAs targeting PCIF1 resulted in a significant increase in *RAB23* mRNA expression and a decrease in *CNOT6* mRNA expression (Figure 2B,C). Immunoblotting analysis showed that the protein expression levels of RAB23 and CNOT6 changed in a similar manner as the mRNA levels (Figure 2D,E). These findings suggest that PCIF1 negatively regulates *RAB23* expression and positively regulates *CNOT6* expression at both the mRNA and protein levels.

**Figure 3:**
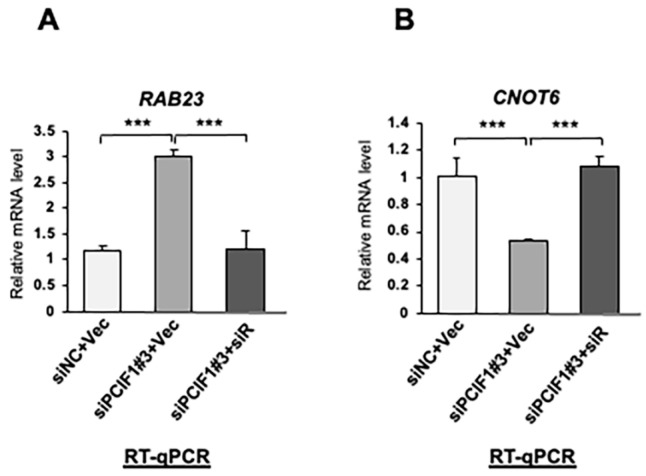
Ectopic expression of siRNA-resistant PCIF1 restores the normal levels of target mRNA expression. (**A**,**B**) RT-qPCR analysis of total RNAs isolated from HeLa cells transfected with a control empty vector (Vec) or a vector expressing siRNA-resistant PCIF1 (PCIF1siR) under treatment with control siRNA (siNC) or PCIF1-targeted siRNAs (siPCIF1 #3), using the specific primer set detecting *RAB23* (**A**) and *CNOT6* (**B**) expression. The *y*-axis represents the fold change relative to the levels in HeLa cells treated with control siRNA. Data are expressed as the mean ± standard deviation of three independent experiments. Asterisks represent statistically significant differences between indicated pairs (Student’s *t*-test, *** *p* < 0.001).

To further eliminate the possibility that the differential expression of these genes by PCIF1 suppression was due to the off-target effects of siRNAs, we performed rescue experiments by exogenously expressing siRNA-resistant PCIF1 under siRNA-mediated PCIF1 knockdown conditions. We transfected a mock vector or rescue vector encoding siRNA-resistant PCIF1 mRNA into HeLa cells to express exogenous PCIF1 at the same level as endogenous PCIF1. By analyzing the mRNA expression of *RAB23* and *CNOT6* using RT-qPCR in the three cell types treated with control knockdown (siNC + Vec), PCIF1 knockdown with a mock vector (siPCIF1#3 + Vec), or PCIF1 knockdown with the siRNA-resistant PCIF1-expressing vector (siPCIF1#3 + siR), we found that the mRNA expression levels of both genes were completely restored to their original levels by the expression of siRNA-resistant PCIF1 mRNA (Figure 3A,B). These results confirm siRNA target specificity and clearly indicate that PCIF1 is responsible for the regulation of these genes.

We further investigated whether the PCIF1-mediated regulation of these target mRNAs could be observed in other human cell lines. We detected similar expression changes in both *RAB23* and *CNOT6* mRNAs upon PCIF1 suppression by the two siRNAs in 293T, MCF7, and Huh7 cells, although the degree of alteration in these cells was lower than that in HeLa cells (Supplementary Figure S5). These results suggest that PCIF1-mediated regulation of *RAB23* and *CNOT6* mRNAs is independent of the cell type.

### 3.3. PCIF1 Specifically Localizes to the Promoter Regions of RAB23 and CNOT6 Genes

**Figure 4:**
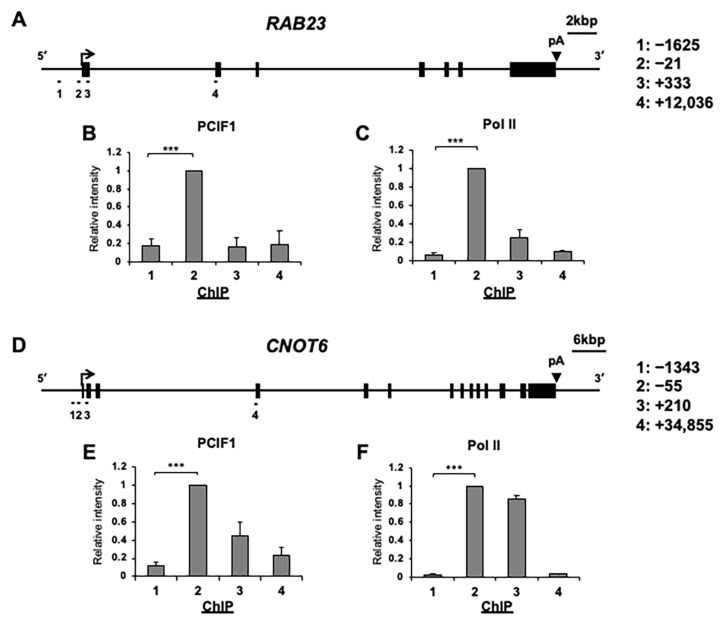
PCIF1 is located at the promoter regions of its target genes. Chromatin immunoprecipitation (ChIP) analyses of *RAB23* (**A**–**C**) and *CNOT6* (**D**–**F**) genes. (**A**,**D**) Schematic representations of the indicated gene structures; the transcription start sites are indicated by arrows, the exons are shown as black boxes, and the polyadenylation signals are indicated by arrowheads. The positions of the PCR amplicons for ChIP are indicated by the numbers below each diagram. The position of the center of each amplicon relative to the transcription start site (+1) is indicated on the right. The lower panel shows the results of the ChIP analyses of the indicated regions of the *RAB23* (**B**,**C**) and *CNOT6* (**E**,**F**) genes in HeLa cells using antibodies against PCIF1 (**B**,**E**) and Pol II (**C**,**F**). The *y*-axis represents the ChIP signal relative to promoter (number 2 position). Data are expressed as the mean ± standard deviation of three independent experiments. Asterisks represent statistically significant differences between position 1 and position 2 (Student’s *t*-test, *** *p* < 0.001).

Next, we explored how PCIF1 regulates the mRNA expression of *RAB23* and *CNOT6*. Previous studies demonstrated that PCIF1 is recruited to the promoters of multiple genes transcribed by RNA polymerase II (Pol II) in a manner dependent on transcriptional activity [25]. Thus, we first determined whether PCIF1 is localized in the *RAB23* and *CNOT6* genes, and if so, which region it localizes to these genes. To test this hypothesis, we conducted ChIP-qPCR experiments to assess the distribution of PCIF1 and Pol II at four different locations of each gene (Figure 4A,D). The results showed that PCIF1 was specifically localized to the promoter regions of both *RAB23* and *CNOT6* (Figure 4B,E), and its distribution pattern was similar to that of Pol II (Figure 4C,F). These results suggest that PCIF1 directly regulates the expression of *RAB23* and *CNOT6* by binding to their promoters.

### 3.4. PCIF1 Does Not Regulate Target Gene Expression at the Transcriptional Level

**Figure 5:**
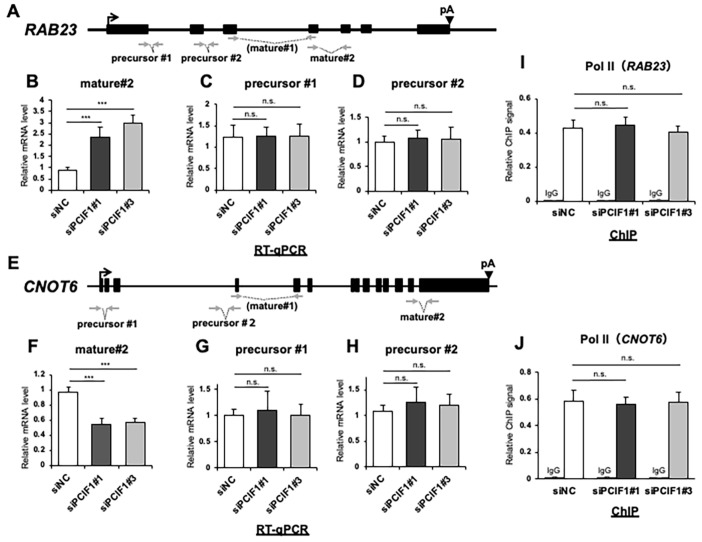
PCIF1 does not regulate expression of its target genes at the transcriptional level. (**A**,**E**) Schematic illustrations of the *RAB23* (**A**) and *CNOT6* (**E**) genes. The transcription start sites are indicated by arrows, the exons are shown as black boxes, and the polyadenylation signals are indicated by arrowheads. The positions of the PCR primer set for RT-qPCR amplification of the precursor and mature mRNAs are indicated by arrows. (**B**–**D**,**F**–**H**) RT-qPCR analysis of total RNAs isolated from HeLa cells treated with control siRNA (siNC) and two distinct PCIF1-targeted siRNAs (siPCIF1 #1 and #3) using the indicated primer sets. (**I**,**J**) ChIP analyses of the *RAB23* (**I**) and *CNOT6* (**J**) gene promoters (position 2 in Figure 4) using antibodies against Pol II in HeLa cells treated with control siRNA (siNC) and two distinct PCIF1 targeted siRNAs (siPCIF1 #1 and #3). Normal rabbit IgG was used as the negative control. Data are expressed as the mean ± standard deviation of three independent experiments. Asterisks represent statistically significant differences between the control siRNA treatment and the indicated PCIF1 targeted siRNA (Student’s *t*-test, n.s. *p* > 0.05, *** *p* < 0.001).

PCIF1 was originally identified as a nuclear protein that binds to the phosphorylated C-terminal domain (CTD) of Pol II [28]. We also found that PCIF1 suppressed the expression of reporter genes driven by various transcriptional activation domains [27,29]. Furthermore, Sendinc et al. reported that PCIF1 depletion altered the transcription of a subset of genes [19]. These observations, along with the promoter localization of PCIF1 on the target genes, suggest that PCIF1 may play a regulatory role in transcription by Pol II. To test whether PCIF1 regulates the transcription of target genes, we first examined whether PCIF1 knockdown affects the expression of precursor forms of target mRNAs. To assess the mRNA precursor levels, we performed RT-qPCR using primer sets that amplify the exon–intron boundary. We used two primer sets per gene to amplify two independent exon–intron boundaries (precursors #1 and #2 in Figure 5A,E). Consistent with the results shown in Figure 2, the expression of mature mRNA (mature#2) was significantly altered in both *RAB23* and *CNOT6* upon PCIF1 knockdown (Figure 5B,F). However, the expression of mRNA precursors (precursor #1 and precursor #2) was not altered (Figure 5C,D,G,H).

To further determine whether PCIF1 plays a role in the initiation of transcription of target genes, we examined the impact of PCIF1 knockdown on the recruitment of Pol II to the promoters of target genes using ChIP-qPCR. Our analyses revealed that PCIF1 knockdown did not significantly alter the distribution of Pol II at *RAB23* and *CNOT6* promoters (Figure 5I,J). These findings indicate that PCIF1 regulates the expression of these target genes at the post-transcriptional level rather than at the transcriptional level.

### 3.5. PCIF1 Regulates Target mRNAs Stability

**Figure 6:**
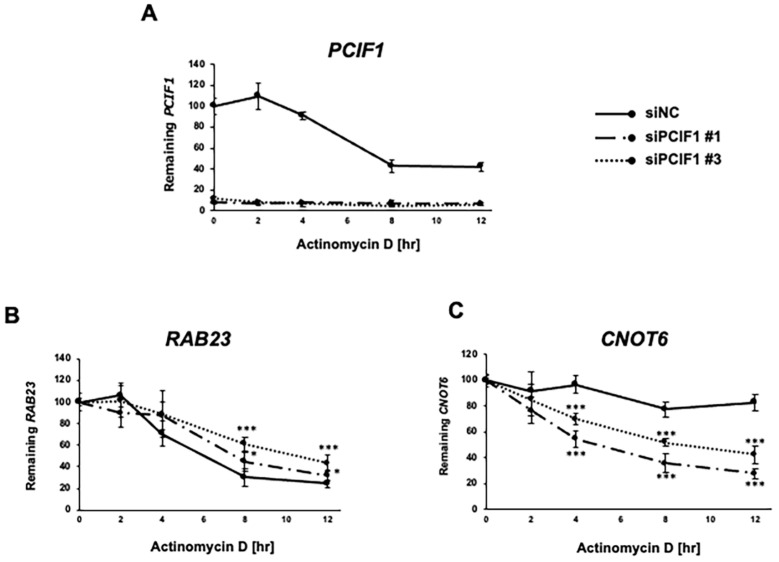
PCIF1 regulates the stability of target gene mRNAs in opposite ways. After HeLa cells were treated with a negative control siRNA (siNC) and two PCIF1-targeted siRNAs (siPCIF1 #1 and #3) for 72 h, actinomycin D was added to inhibit transcription. Cells were harvested at 0, 2, 4, 8, and 12 h after treatment, and total RNA was isolated. The amount of residual mRNA was analyzed by RT-qPCR at each time point, using the specific primer set detecting *PCIF1* (**A**), *CRAB23* (**B**), and *CNOT6* (**C**) mRNAs. The relative value was calculated using the expression level of β-actin mRNA (ACTB) as a normalizer. The relative values at each time point were calculated relative to time zero. Asterisks represent statistically significant differences between the control siRNA treatment and the indicated PCIF1-targeted siRNA (Student’s *t*-test, * *p* < 0.05, *** *p* < 0.001).

To investigate the potential function of PCIF1 to post-transcriptionally regulate target gene expression, we examined the effect of PCIF1 knockdown on target mRNAs’ stability. Following actinomycin D treatment to inhibit transcription under PCIF1 knockdown conditions, RNA was collected at various time points, and the remaining amount of target mRNAs was analyzed by RT-qPCR. As shown in Figure 6A, treatment of HeLa cells with the two siRNAs resulted in efficient knockdown of PCIF1. Upon PCIF1 knockdown, there was a significant increase in the half-life of *RAB23* mRNA from 6 h to 8–12 h (Figure 6B). Conversely, PCIF1 depletion led to a significant decrease in the stability of *CNOT6* mRNA from over 12 h to 6–10 h (Figure 6C). These results suggest that PCIF1 negatively regulates the stability of *RAB23* mRNA but positively regulates that of *CNOT6* mRNA.

### 3.6. PCIF1 Regulates Target Gene Expression in an RNA Methylation-Dependent Manner

**Figure 7:**
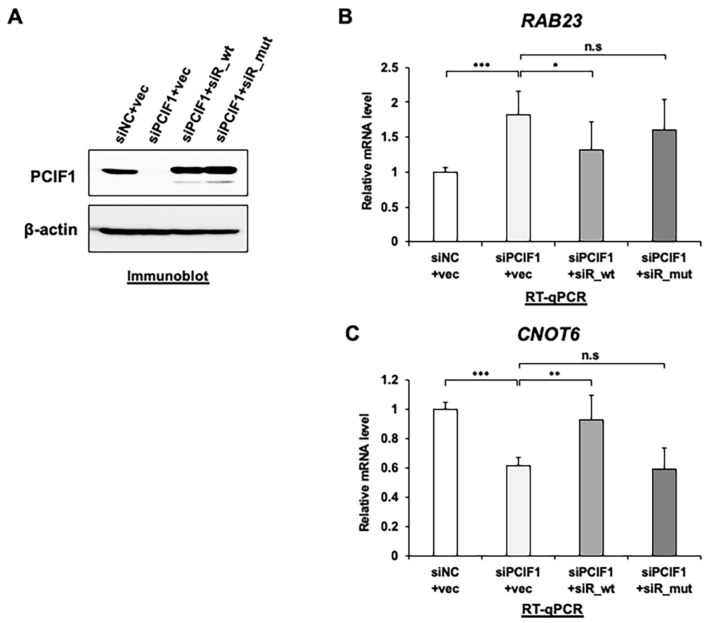
Ectopic expression of siRNA-resistant wild-type but not methyltransferase-deficient mutant PCIF1 restored normal levels of target mRNA expression. (**A**) Immunoblotting analysis of total protein extracts from HeLa cells transfected with a control empty vector (Vec), a vector expressing siRNA-resistant wild-type PCIF1 (siR_wt), or methyltransferase-deficient mutant PCIF1 (siR_mut) under treatment with control siRNA (siNC) or PCIF1-targeted siRNAs (siPCIF1: siPCIF1 #3 was used for PCIF1 suppression) with the indicated antibodies. (**B**,**C**) RT-qPCR analysis of total RNAs isolated from HeLa cells treated as in (**A**), using the specific primer set detecting *RAB23* (**B**) and *CNOT6* (**C**) expression. Data are expressed as the mean ± standard deviation of three independent experiments. Asterisks represent statistically significant differences between the indicated pairs (Student’s *t*-test, n.s. *p* > 0.05, * *p* < 0.05, ** *p* < 0.01, *** *p* < 0.001).

Subsequently, we examined whether the methyltransferase activity of PCIF1 is involved in the regulation of target gene mRNA expression. To test this, we conducted rescue experiments in which siRNA-resistant PCIF1 wild-type or methyltransferase activity-deficient PCIF1 mutant were expressed under knockdown conditions. We developed a PCIF1 mutant in which the catalytic NPPF motif was replaced with NAAF, the same mutant that we previously used for in vitro methylation activity [16]. We first knocked down endogenous PCIF1 using siRNA and then transfected either the wild-type or methyltransferase activity-deficient mutant PCIF1 construct into knockdown cells to express exogenous PCIF1 at the same level as endogenous PCIF1 (Figure 7A). Consistent with the results shown in Figure 3, the expression of PCIF1 wild-type resulted in the restoration of the mRNA expression of both target genes (siPCIF1 + siR_wt in Figure 7B,C). In contrast, the expression of the methyltransferase activity-deficient mutant did not restore the mRNA expression of the target genes (siPCIF1 + siR_mut in Figure 7B,C). These results suggest that the RNA methyltransferase activity of PCIF1 is required for the regulation of *RAB23* and *CNOT6* expression.

### 3.7. PCIF1 Suppression Resulted in a Significant Decrease in m^6^A Levels in Both RAB23 and CNOT6 mRNAs

**Figure 8:**
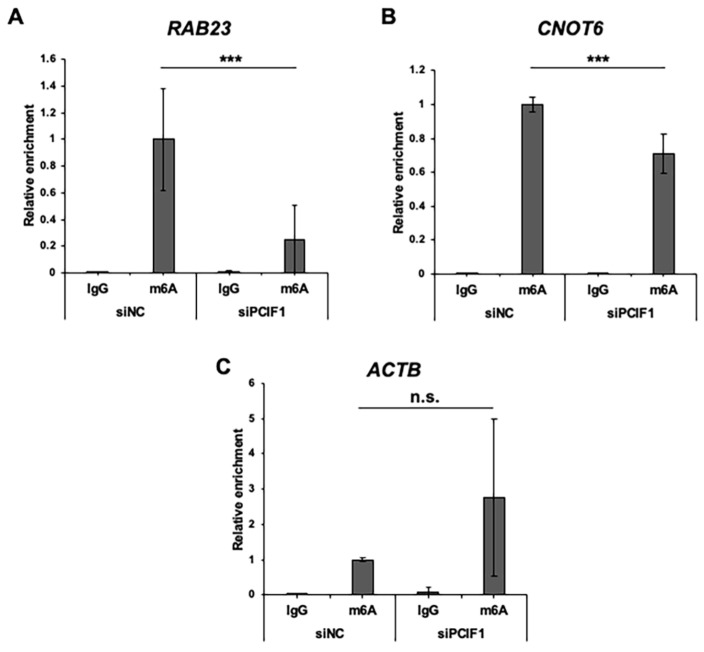
PCIF1 suppression resulted in a significant decrease in m^6^A levels of both *RAB23* and *CNOT6* mRNAs. MeRIP-qPCR analysis was performed using HeLa cells treated with control siRNA (siNC) or PCIF1-targeted siRNA (siPCIF1: siPCIF1 #3 was used for PCIF1 suppression). RT-qPCR analysis of RNAs purified from the immunoprecipitates of HeLa cell extracts using the anti-m^6^A antibody, using the specific primer set detecting *RAB23* (**A**), *CNOT6* (**B**), and *ACTB* (**C**) mRNAs. The *y*-axis represents the fold change relative to the RNA levels in the immunoprecipitate by anti-m^6^A antibody from HeLa cells treated with control siRNA (siNC). Normal rabbit IgG (IgG) was used as a negative control. Data are expressed as the mean ± standard deviation of three independent experiments. Asterisks represent statistically significant differences between the indicated pairs (Student’s *t*-test, n.s. *p* > 0.05, *** *p* < 0.001).

The regulation of *RAB23* and *CNOT6* mRNAs by PCIF1 through methyltransferase activity suggests that these mRNAs are modified by m^6^Am via PCIF1. To determine whether m^6^A levels of these target mRNAs were affected by PCIF1 suppression in HeLa cells, we conducted MeRIP-qPCR analysis. Total RNAs from control or PCIF1 knockdown cells were immunoprecipitated with an anti-m^6^A antibody, and *RAB23* and *CNOT6* mRNAs in the coprecipitated RNAs were analyzed by RT-qPCRs. The results showed that PCIF1 suppression resulted in a significant decrease in m^6^A levels in both *RAB23* and *CNOT6* mRNAs compared to control knockdown (Figure 8A,B). In contrast, m^6^A levels in β-actin mRNA were not significantly altered (Figure 8C). Together with the results shown in Figure 7, these findings suggest that PCIF1 regulates the stability of *RAB23* and *CNOT6* mRNAs through m^6^Am modification in opposite way.

## 4. Discussion

The m^7^G cap-adjacent m^6^Am modification in mammalian and viral mRNAs was discovered almost 50 years ago [12]. Due to its prevalence, this modification has been hypothesized to play a crucial role in the regulation of mRNA metabolism and function [12,13,23]. The recent discovery that PCIF1 is the sole m^6^A enzyme responsible for m^6^Am has promoted functional and mechanistic studies of m^6^Am in gene expression. However, conflicting findings regarding the role of m^6^Am in mRNA stability and protein translation have been reported in multiple studies using PCIF1 knockout cells [16,17,18,19,24,30]. These discrepancies may be attributed to differences in cell types and methodologies used. Additionally, we speculate that the controversy may be partially attributed to the indirect effects of PCIF1 loss and context-dependent changes in gene expression caused by compensatory responses triggered by PCIF1 knockout.

To circumvent these issues, we employed an siRNA-mediated knockdown approach to examine the effects of PCIF1 depletion within a shorter time frame. We successfully identified a subset of differentially expressed genes (DEGs) with limited overlap with previously identified DEGs under PCIF1 knockout conditions. After eliminating the possible off-target effects of siRNAs, *RAB23* and *CNOT6* were identified as novel and accurate PCIF1 targets. We further demonstrated that PCIF1 regulates the stability of these mRNAs in opposite ways through m^6^A modification via methyltransferase activity. Although we utilized the siRNA-mediated transient knockdown method to deplete PCIF1, acute endogenous protein degradation methodologies such as degron-based systems are superior for observing the direct effects of PCIF1 repression on gene expression within a shorter time frame (typically several hours) [31]. Therefore, in the future, we will analyze the direct effects of PCIF1 depletion on gene expression using these degron-based systems.

Two recent genome-wide m^6^Am site-mapping studies have reported that both *RAB23* and *CNOT6* mRNAs contain m^6^Am modifications in Calu-3 and 293T cells, respectively [30,32]. Consequently, together with our MeRIP-qPCR assays (Figure 8), we predicted that these target mRNAs would be modified by m^6^Am in HeLa cells. However, our MeRIP assay could not distinguish between cap-adjacent m^6^Am and internal m^6^A. Therefore, it is possible that PCIF1 suppression reduced internal m^6^A levels in these target mRNAs. Notably, several recent genome-wide m^6^A mapping studies have shown that PCIF1 depletion results in a significant reduction in internal m^6^A levels at specific sites in certain genes [33,34]. These possible PCIF1-dependent internal m^6^A modifications may be mediated by non-canonical PCIF1 activity on internal adenosine or indirectly by the canonical m^6^A methyltransferase METTL3/14 complex. However, a recent biochemical study using recombinant PCIF1 and in vitro synthesized RNA substrates demonstrated that the methyltransferase activity of PCIF1 on internal adenosine is marginal, suggesting that direct modification of internal m^6^A is less likely [35]. Therefore, it would be interesting to test whether PCIF1 suppression affects the activity of the METTL3/14 complex on specific m^6^A sites.

Although we identified *RAB23* and *CNOT6* as novel PCIF1 targets, it is unclear which biological process is affected by the PCIF1-mediated regulation of these target mRNAs. *RAB23* encodes a small GTPase that plays a crucial role in intracellular membrane trafficking and protein transport [36]. Additionally, it functions as a negative regulator of the sonic hedgehog (Shh) signaling pathway [37]. Genetic mutations in *RAB23* can cause Carpenter syndrome (CS), a rare autosomal recessive disorder characterized by craniofacial defects, polydactyly, cardiac defects, craniosynostosis, and obesity [38]. RAB23 also localizes to cilia and promotes cilium formation by regulating protein trafficking and turnover within the primary cilium [39]. Interestingly, a recent study reported that PCIF1 functions as a negative regulator of cilia formation in human RPE-1 cells by reducing mRNA stability and translation of *BICD2* mRNA through its m^6^Am methyltransferase activity [40]. Although it is unknown whether PCIF1 also inhibits *RAB23* expression in RPE-1 cells, the negative regulation of *RAB23* by PCIF1 may contribute to the inhibitory effect of PCIF1 on ciliogenesis. We are currently testing whether PCIF1 is involved in RAB23-mediated cellular processes and diseases.

CNOT6 possesses 3′–5′ RNase activity on polyadenylated RNA substrates and is a catalytic subunit of the CCR4-NOT complex, which is involved in the regulation of mRNA degradation, translation, and transcription [41]. Although CNOT6 depletion appears to have a relatively minor impact on mRNA metabolism due to the compensatory role of its paralog CNOT6L, CNOT6 repression can still result in increased stability of certain mRNAs [42]. PCIF1 depletion significantly reduced *CNOT6* mRNA and protein levels, suggesting that some of the mRNAs upregulated by PCIF1 depletion identified in this study may not be direct targets of PCIF1 but rather may be directly regulated by CNOT6. In this regard, it is possible that *RAB23* mRNA may be indirectly regulated by PCIF1 through a decrease in CNOT6 by PCIF1 repression. However, we found that the methylation level of *RAB23* mRNA was markedly reduced by PCIF1 repression (Figure 8), suggesting that PCIF1-mediated methylation directly regulates *RAB23* mRNA stability.

Although m^6^Am modifications were initially reported to contribute to mRNA stabilization, recent studies with more specific genome-wide mapping of m^6^Am sites have shown that m^6^Am does not affect mRNA stability [19,30,34], raising questions about the role of m^6^Am in stabilizing mRNAs. However, the present study demonstrated that PCIF1-mediated m^6^Am modification is involved in regulating the stability of specific mRNAs. This result is consistent with those of multiple recent studies. Zhang et al. reported that PCIF1 suppresses HIV infection by stabilizing the mRNA of ETS1 transcription factor, which inhibits the transcription of viral genes through m^6^Am modification [33]. In addition, Wang et al. reported that PCIF1 promotes SARS-CoV-2 infection by stabilizing mRNAs encoding host receptors for coronavirus infection, such as *ACE2* and *TMPRSS2* through m^6^Am modification [32], suggesting that PCIF1 and m^6^Am are potential therapeutic targets for preventing coronavirus infection. Furthermore, the involvement of the PCIF1-mediated m^6^Am modification in cancer progression has been suggested in several human cancers. For example, PCIF1 is involved in gastric cancer progression by inhibiting the mRNA translation of the tumor suppressor gene *TM9SF1* via m^6^Am modification [34]. Additionally, PCIF1 has been reported to regulate colon cancer growth and response to anti-PD-1 by stabilizing cancer-promoting gene mRNAs, such as *FOS*, *IFITM3*, and *STAT1*, through m^6^Am modifications [43]. These studies clearly demonstrate the stabilizing effect of PCIF1-mediated m^6^Am modification on specific mRNAs.

The results of this study suggest that PCIF1 regulates the stability of *RAB23* and *CNOT6* mRNAs in opposing ways through m^6^A modifications. How does PCIF1 regulate mRNA stability in opposing ways depending on the gene? One of the major mechanisms regulating mRNA stability is decapping, which removes the cap structure of mRNAs and plays an important role in the mRNA degradation pathway. Decapping is catalyzed by decapping enzymes. The recruitment and activity of decapping enzymes are affected by the sequence and structure near the 5′-end and RNA-binding proteins [44,45]. Therefore, m^6^Am modifications adjacent to the cap may also affect cap recognition and decapping enzyme activity. A mechanism by which m^6^Am modification inhibits mRNA degradation by blocking cap recognition of the decapping enzyme Dcp2 has been proposed [20]. However, a recent study reported that neither 2′-*O*-methylation nor *N*^6^-methylation of adenosine inhibits RNA decapping by Dcp2 in vitro [46]. Thus, it is still unclear whether RNA sensitivity to decapping by Dcp2 is affected by m^6^Am modification.

Notably, there are many other decapping enzymes in mammals besides Dcp2, including Nudt16 and Nudt3, each of which targets a specific subset of mRNAs and may regulate decapping through different mechanisms [44,45]. It has also been suggested that Dcp2 functions preferentially on unstable mRNAs, whereas Nudt16 functions on longer-lived mRNAs [47]. Interestingly, our results showed that *RAB23* mRNA has a relatively short half-life, whereas *CNOT6* mRNA is very stable (Figure 6). These findings suggest that *RAB23* and *CNOT6* mRNAs may be targets of different decapping enzymes. Indeed, *RAB23* mRNA has been reported to be a target gene of Dcp2 because its expression is upregulated by Dcp2 repression [48]. Therefore, we postulate that m^6^Am modification of *RAB23* mRNA may promote mRNA degradation by facilitating cap recognition and decapping by Dcp2 through a mechanism defined by a combination of the specific sequence, structure, RNA-binding proteins, and m^6^Am modifications. In contrast, we postulate that m^6^Am modification of the more stable *CNOT6* mRNA suppresses mRNA degradation by inhibiting cap recognition by Nudt16, another decapping enzyme, through a mechanism involving both m^6^Am modification and specific sequences. The molecular mechanisms underlying the target specificity of each decapping enzyme and the distinct mechanisms by which each enzyme is involved remain unknown. Future studies are needed to analyze how cap recognition and activity of each decapping enzyme are affected by m^6^Am modification. It is also necessary to explore whether m^6^Am-binding proteins (m^6^Am readers) and mRNA-specific binding proteins cooperatively regulate the decapping activity.

Previous studies have shown that deletion of PCIF1 has only minor effects on normal growth and development of cultured cells and mice [16,24,29,49,50]. However, recent studies have provided evidence that PCIF1-mediated m^6^Am modification is involved in a variety of biological processes, including stress response [16,30], viral infection [32,33,51], cancer [34,43,52,53,54], metabolism [55], and ciliogenesis [40]. Thus, PCIF1 and m^6^Am may be particularly important for the regulation of gene expression in response to extracellular stimuli and differentiation signals. Therefore, it is important to identify specific stimulus-dependent targets of PCIF1. Further studies are needed to elucidate the molecular mechanisms underlying PCIF1-mediated regulation of these target genes, which will contribute to our understanding of the roles of m^6^Am and PCIF1 in specific biological processes and diseases.

## 5. Conclusions

In this study, we investigated the effects of siRNA-mediated transient suppression of PCIF1, the sole enzyme responsible for *N*^6^,2′-*O*-dimethyladenosine (m^6^Am) formation, on global mRNA expression in HeLa cells and identified a subset of DEGs. After excluding the possible off-target effects of siRNAs, *RAB23* and *CNOT6* were identified as accurate PCIF1 targets. Upon PCIF1 suppression, RAB23 expression increased, whereas CNOT6 expression decreased at both the mRNA and protein levels. We found that PCIF1 regulates the stability of these mRNAs in opposite ways through m^6^A modification via methyltransferase activity.

## Figures and Tables

**Table 1:** Pathway analysis of the downregulated genes.

Molecular and Cellular Functions	*p*-Value	No. of Molecules
Cellular Movement	4.65 × 10^−5^	16
Lipid Metabolism	1.93 × 10^−4^	11
Small Molecule Biochemistry	1.93 × 10^−4^	16
Cell Signaling	3.81 × 10^−4^	6
Nucleic Acid Metabolism	3.81 × 10^−4^	5
**Physiological System Development and Function**	***p*-value**	**No. of Molecules**
Tissue Morphology	2.04 × 10^−5^	15
Hematological System Development and Function	8.24 × 10^−4^	14
Hematopoiesis	8.24 × 10^−4^	5
Humoral Immune Response	8.24 × 10^−4^	3
Lymphoid Tissue Structure and Development	8.24 × 10^−4^	4

Pathway analysis of downregulated genes using Ingenuity Pathway Analysis (IPA) software version 11.0. Based on the *p*-values, each top-five category for “molecular and cellular function” and “physiological system development and function” are listed.

**Table 2:** Pathway analysis of the upregulated genes.

Molecular and Cellular Functions	*p*-Value	No. of Molecules
Molecular Transport	8.10 × 10^−4^	15
Nucleic Acid Metabolism	8.10 × 10^−4^	10
Small Molecule Biochemistry	8.10 × 10^−4^	23
Amino Acid Metabolism	1.01 × 10^−3^	3
Cellular Movement	1.41 × 10^−3^	18
**Physiological System Development and Function**	***p*-value**	**No. of Molecules**
Hematological System Development and Function	1.90 × 10^−4^	15
Immune Cell Trafficking	1.90 × 10^−4^	10
Tissue Development	1.90 × 10^−4^	15
Cardiovascular System Development and Function	1.23 × 10^−3^	12
Organismal Development	1.23 × 10^−3^	13

Pathway analysis of upregulated genes using Ingenuity Pathway Analysis (IPA) software version 11.0. Based on the *p*-values, each top-five category for “molecular and cellular function” and “physiological system development and function” are listed.

## Data Availability

DNA microarray data were deposited in the Gene Expression Omnibus under the accession number GSE156768. All other data that support the findings of this study are available from the corresponding author Y.H. upon reasonable request.

## Supplementary Materials

The following supporting information can be downloaded at: https://www.mdpi.com/article/10.3390/cells13201689/s1, Figure S1: The effect of siRNA-mediated PCIF1 suppression on the mRNA expression of selected upregulated genes identified by microarray analysis, Figure S2: The effect of siRNA-mediated PCIF1 suppression on the mRNA expression of selected downregulated genes identified by microarray analysis, Figure S3: PCIF1-mediated regulation of *RAB23* and *CNOT6* mRNAs is independent of the cell type, Figure S4: The effect of siRNA-mediated PCIF1 suppression on the mRNA expression of selected downregulated genes identified by microarray analysis, Figure S5: PCIF1-mediated regulation of *RAB23* and *CNOT6* mRNAs is independent of the cell type, Supplemental document S1: Supplemental Figure legends and primer sequences.
