# Supplementary material for: Cap-Specific m6Am Methyltransferase PCIF1/CAPAM Regulates mRNA Stability of RAB23 and CNOT6 through the m6A Methyltransferase Activity
Source: Cells. 2024 Oct 12;13(20):1689. doi: 10.3390/cells13201689 (PMC11506431; doi:10.3390/cells13201689)
Supplement: Supplementary file 1 [file cells-13-01689-s001.zip › Supplemental document S1.pdf]

Supplemental Figure legends

Figure S1: The effect of siRNA-mediated PCIF1 suppression on the mRNA expression of selected upregulated genes identified by microarray analysis.

RT-qPCR analysis of total RNAs isolated from HeLa cells transfected with a negative control siRNA (siNC) or two distinct PCIF1 targeted siRNAs (siPCIF1 #1 and #2), using the primer sets for indicated upregulated genes. The y-axis represents the fold-change relative to the levels in HeLa cells treated with control siRNA. Data are expressed as the mean ± standard deviation of three independent experiments.

Figure S2: The effect of siRNA-mediated PCIF1 suppression on the mRNA expression of selected upregulated genes identified by microarray analysis.

RT-qPCR analysis of total RNAs isolated from HeLa cells transfected with a negative control siRNA (siNC) or two distinct PCIF1 targeted siRNAs (siPCIF1 #1 and #2), using the primer sets for indicated downregulated genes. The y-axis represents the fold-change relative to the levels in HeLa cells treated with control siRNA. Data are expressed as the mean ± standard deviation of three independent experiments.

Figure S3: The effect of siRNA-mediated PCIF1 suppression on the mRNA expression of selected upregulated genes identified by microarray analysis.

RT-qPCR analysis of total RNAs isolated from HeLa cells transfected with a negative control siRNA (siNC) or three distinct PCIF1 targeted siRNAs (siPCIF1 #1, #2, and #3), using the primer sets for *PCIF1* and indicated upregulated genes (*EGR1*, *CA12*, *RAB23*, *PTGES*, and *HMOX1*). The y-axis represents the fold-change relative to the levels in HeLa cells treated with control siRNA. Data are expressed as the mean ± standard deviation of three independent experiments.

Figure S4: The effect of siRNA-mediated PCIF1 suppression on the mRNA expression of selected downregulated genes identified by microarray analysis.

RT-qPCR analysis of total RNAs isolated from HeLa cells transfected with a negative control siRNA (siNC) or three distinct PCIF1 targeted siRNAs (siPCIF1 #1, #2, and #3), using the primer sets for indicated downregulated genes (PCIF1, *PRKA*, *CNOT6*, and *SLC2A3*). The y-axis represents the fold-change relative to the levels in HeLa cells treated with control siRNA. Data are expressed as the mean ± standard deviation of three independent experiments.

Figure S5: PCIF1-mediated regulation of *RAB23* and *CNOT6* mRNAs is independent of the cell type

RT-qPCR analysis of total RNAs isolated from the indicated human cells (HeLa, 293T, MCF7, Huh7) transfected with a negative control siRNA (siNC) or two distinct PCIF1 targeted siRNAs (siPCIF1 #1 and #3), using the primer sets for indicated genes (*PCIF1*, *RAB23*, and *CNOT6*). The y-axis represents the fold-change relative to the levels in each cell line treated with control siRNA. Data are expressed as the mean ± standard deviation of three independent experiments.

Supplemental Experimental Procedures

Primers

The primers used in this study were as follows:

[RT-PCR primers for mature mRNAs]

PCIF1_F 5’-AGCTGGCTTCGGAAGGACCAC-3’

PCIF1_R 5’-GCCACACTGCCTCCGCAGAT-3’

ACTB_F1 5’-AGAAATCTGGCACCACACC-3’

ACTB_R1 5’-TAGCACAGCCTGGATAGCAA-3’

RAB23_F 5’-TGCAGGTCAGGAGGAATTTG-3’

RAB23_R 5’-TCTCCCACTTCGGCTACTACTTTC-3’

RAB23_F2 5’-ACTACAAGAAAACCATTGGAGTTGA-3’

RAB23_R2 5’-GACCTGCAGTGTCCCATAACA-3’

CNOT6_F 5’-GTGCCTATGAGAGTGGCCTGATGC-3’

CNOT6_R 5’-AGGGCCCAGGATGCCTAAGGTG-3’

CNOT6_F2 5’-GAAATCCCACTGGGCAGAGC-3’

CNOT6_R2 5’-AGTGACCACAAAGATGCGCT-3’

r18S_F 5’-GTAACCCGTTGAACCCATT-3’

r18S_R 5’-CCATCCAATCGGTAGTAGCG-3’

[RT-PCR primers for precursor mRNAs]

RAB23 pre_F 5’-CATCGGCAGTTCCCAGGAG-3’

RAB23 pre_R 5’-CCCTACTGCCACTTCACTAGC-3’

RAB23 pre2_F 5’-TTTTTGGAGCGACAAATTCAG-3

RAB23 pre2_R 5’-TTCACAAATCAAAGCCAAAGGA-3’

CNOT6 pre_F 5’-TCTTAACAGGCATGCCCAAA-3’

CNOT6 pre_R5’-TTCCATTTGCTGCTTCCTCA-3’

CNOT6 pre2_F 5’-ACCCAAATTGCATCCCTCCT-3’

CNOT6 pre2_R 5’-TTAGCAGGCTCTTCGGGGAA-3’

[primers for ChIP assay]

RAB23_ChIP1_F 5’-GTTGCTTTCTGCCGTGTG-3’

RAB23_ChIP1_R 5’-GGTCTGCTGCCAACCTT-3’

RAB23_ChIP2_F 5’-CTCCTGTTCGACAGTCAGC-3’

RAB23_ChIP2_R 5’-TTCAGGCCGTCCCTAGC-3’

RAB23_ChIP3_F 5’-CAGAAACAGGAGGTCCCTACT-3’

RAB23_ChIP3_R 5’-AGCGCGAAAGGAAAGAAAGC-3’

RAB23_ChIP4_F 5’-ATAGGCGAGATCCCTCCAA-3’

RAB23_ChIP4_R 5’-TGAAGACGCCAGTGGAC-3’

CNOT6_ChIP1_F 5’-AAGACGCTTTGCAGCAAAATC-3’

CNOT6_ChIP1_R 5’-AGGCCTTTGCCGCAAAC-3’

CNOT6_ChIP2_F 5’-GGAGGGATCGCGCTGAGTA-3’

CNOT6_ChIP2_R 5’-TCTGCCTCTCGCTGGAATTAC-3’

CNOT6_ChIP3_F 5’-CGGGTAGTGGAAAACCAGGTAA-3’

CNOT6_ChIP3_R 5’-TCGACTCATCTCAGCATTAAAGTGA-3’

CNOT6_ChIP4_F 5’-CGGCTGGATACCTTTCCCAT-3’

CNOT6_ChIP4_R 5’-TTCCTAATAAGAGTGGCCCGTTAA-3’

[primers for mutagenesis]

PCIF1_siR_mF 5’-AGCCCCAGTATGTTCCGTGAAATCATGAACGACATTCC-3’

PCIF1_siR_R 5’-CACGACTGGTTCACAGTTGCTGGGC-3’

PCIF1_PF-AA_F 5’-GCCGCCTGCGAGGAGCTCATGGATGCCA-3’

PCIF1_P554_R 5’-AGGGTTGGCCTCAAATGAACCACTC-3’The primers used in the

The nucleotide sequences of the RT-qPCR primers used in the Supplemental Figures are available upon request.
