# Supplementary figures and images for: Cap-Specific m6Am Methyltransferase PCIF1/CAPAM Regulates mRNA Stability of RAB23 and CNOT6 through the m6A Methyltransferase Activity
Source: Cells. 2024 Oct 12;13(20):1689. doi: 10.3390/cells13201689 (PMC11506431; doi:10.3390/cells13201689)

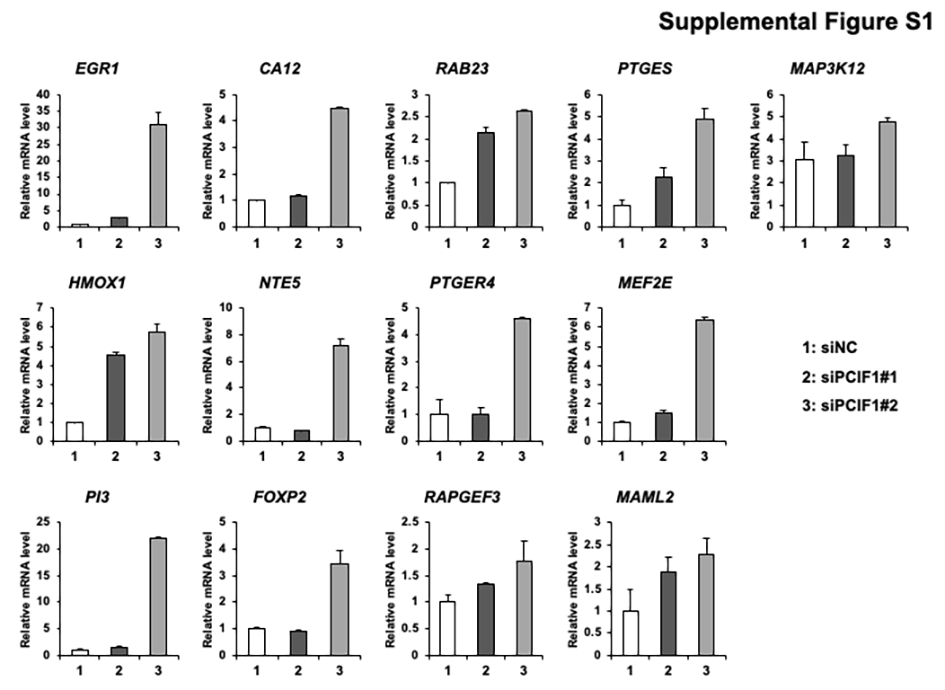

Supplement: Supplementary file 1 [file cells-13-01689-s001.zip › Sugita_PCIF1-2_Figure S1.tiff]

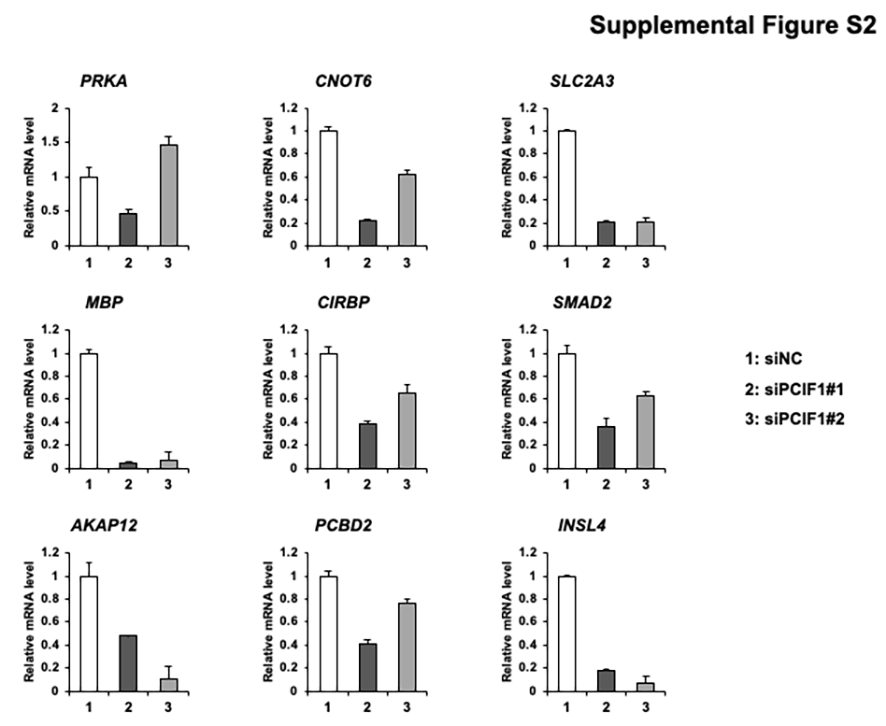

Supplement: Supplementary file 1 [file cells-13-01689-s001.zip › Sugita_PCIF1-2_Figure S2.tiff]

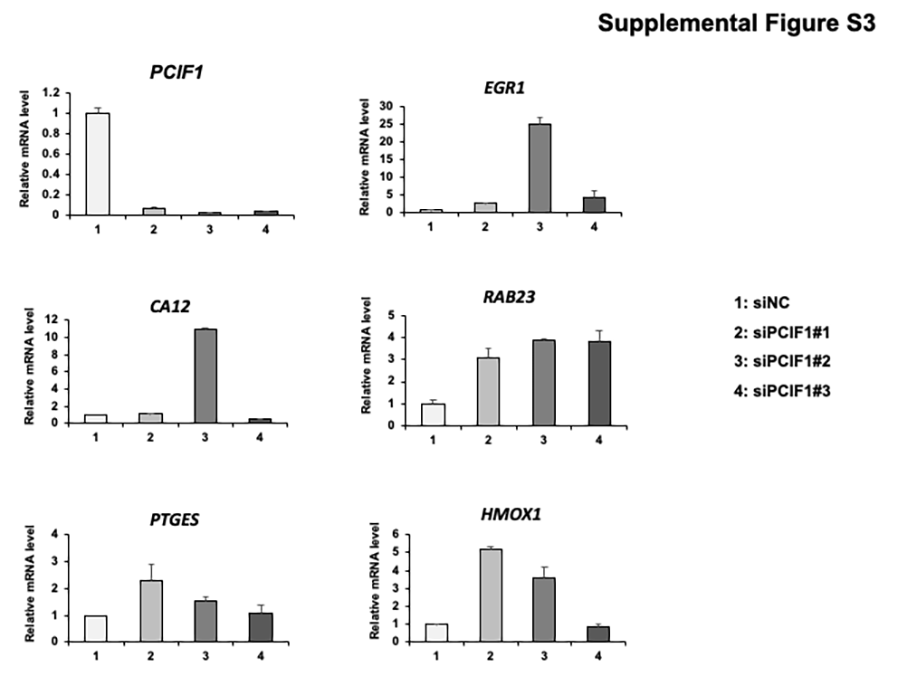

Supplement: Supplementary file 1 [file cells-13-01689-s001.zip › Sugita_PCIF1-2_Figure S3.tiff]

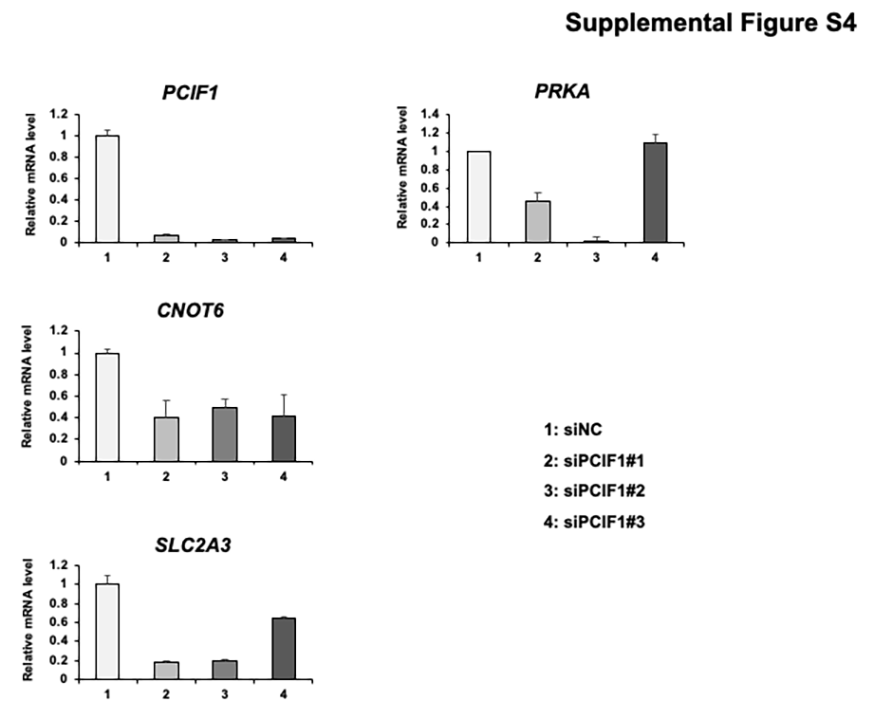

Supplement: Supplementary file 1 [file cells-13-01689-s001.zip › Sugita_PCIF1-2_Figure S4.tiff]

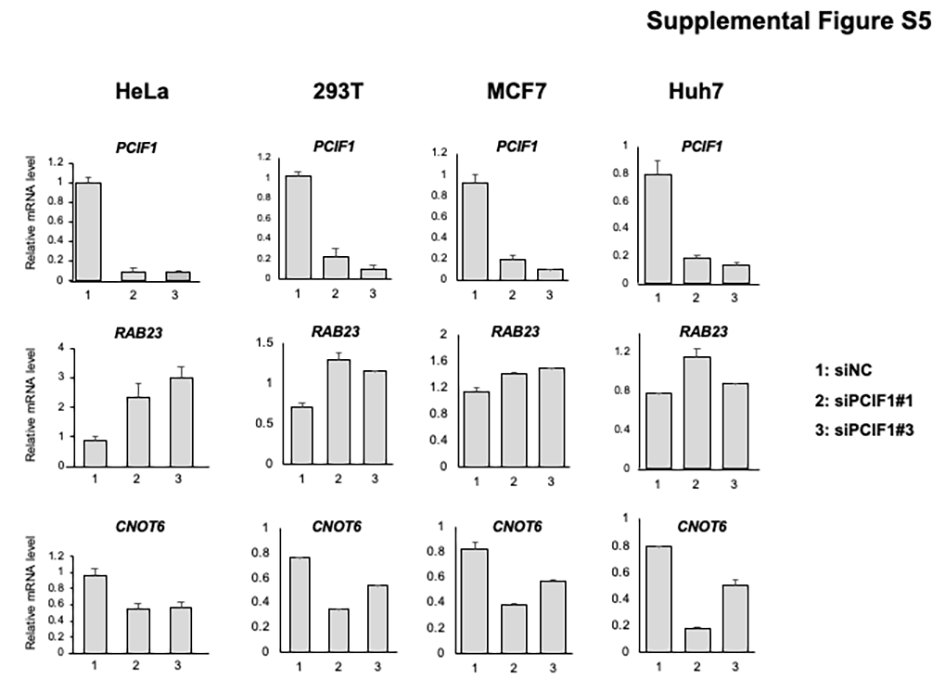

Supplement: Supplementary file 1 [file cells-13-01689-s001.zip › Sugita_PCIF1-2_Figure S5.tiff]
